# High hydrostatic pressure shapes the development and production of secondary metabolites of Mariana Trench sediment fungi

**DOI:** 10.1038/s41598-021-90920-1

**Published:** 2021-06-01

**Authors:** Qingqing Peng, Yongqi Li, Ludan Deng, Jiasong Fang, Xi Yu

**Affiliations:** Shanghai Engineering Research Center of Hadal Science and Technology, College of Marine Sciences, Shanghai Ocean University, Shanghai, China

**Keywords:** Marine biology, Fungi

## Abstract

The hadal biosphere is one of the least understood ecosystems on our planet. Recent studies have revealed diverse and active communities of prokaryotes in hadal sediment. However, there have been few studies on fungi in hadal sediment. Here we report the first isolation and cultivation of 8 fungi from the Mariana Trench sediment. The individual colonies were isolated and identified as *Stemphylium* sp., *Cladosporium* sp., *Arthrinium* sp., *Fusarium* sp*.*, *Alternaria* sp., and *Aspergillus* sp. High hydrostatic pressure (HHP) test was carried out to identify the piezophily of these hadal fungi. Among them, 7 out of the 8 fungal isolates exhibited the ability of germination after incubation under 40 MPa for 7 days. Vegetative growth of the isolates was also affected by HHP. Characterization of secondary metabolites under different pressure conditions was also performed. The production of secondary metabolites was affected by the HHP treatment, improving the potential of discovering novel natural products from hadal fungi. The antibacterial assay revealed the potential of discovering novel natural products. Our results suggest that fungal growth pressure plays an important role in the development and production of secondary metabolites of these hadal fungi under the extreme environment in the Mariana Trench.

## Introduction

The hadal zone, comprised mostly of trenches, is the deepest area of the oceans. Although representing only 1–2% of the global ocean benthic area, the hadal zone constitutes 45% of the ocean depth^1^. Thus, the hadal zone is characterized by high hydrostatic pressure, with other physical and chemical parameters similar to the abyssal oceans^2^. Early studies postulate that the hadal zone is typified by scarce food and low input of organic matter^3^. However, recent studies have shown that the trench sediment had abundant sedimentary organic carbon^4^. Microbiological and biogeochemical investigations showed unexpected high microbial abundance, diversity and activities^5^. Furthermore, a large number of piezotolerant and piezophilic bacteria and archaea, such as *Photobacterium, Shewanella, Thermococcus,* and *Pyrococcus*^6^, have been isolated from trench sediment and other matrices^7^. The mechanisms of microbial adaptation to high hydrostatic pressure (HHP) have been elucidated, such as biosynthesis of unsaturated membrane lipids, transporters, and motility and respiratory chain components^8,9^.

Deep-sea microorganisms are important members of halobios, which can produce novel substances with antibacterial, anti-tumor, anti-protease, and anti-virus activities^10^. Among them, fungi are considered as the most promising drug source for sustainable utilization^11^. According to previous studies, 75% of natural products produced by deep-sea fungi have bioactivity and 40% of those have the potential to be drug candidates^12^. The first fungus isolated from the Mariana Trench was reported by Takami^13^. Since then, a number of fungi species have been isolated from the deep ocean^10,14–19^. More and more bioactive compounds isolated from deep-sea derived fungi have been published^20^. Up until now, there have been only a few reported piezotolerant fungi isolated from the deep ocean, and no piezophilic fungi from the hadal zone have been reported. Some of the deep-sea fungi can grow at pressures up to 20 MPa^21^ and the growth characteristics were described under the simulated deep-sea conditions^15,22^. It was reported that hydrostatic pressure appeared to play a role in the developments of mycelium and spores, as was typical of the genus *Aspergillus*^22^. Several fungal isolates showed abnormal phenotypes under elevated pressure, implying that HHP would change the life process of fungi in the deep sea. Although recent studies have reported diverse fungi from the deep ocean, isolation and cultivation of marine fungi under HHP conditions from the hadal sediment have not been reported thus far. Furthermore, most of the reported fungal secondary metabolites are produced under atmospheric conditions. The novel biosynthetic pathway of secondary metabolites under in-situ environmental conditions is not known. That impedes the exploration of deep-sea life processes and limits the utilization of these natural microbial resources.

In this paper, we report the isolation of sediment fungi from the Mariana Trench, and show that the development and production of secondary metabolites of these fungi are influenced by growth pressure. Our study represents the first report of 8 fungi in the Mariana Trench sediment and the special life processes of the hadal fungi under extreme high pressure conditions.

## Materials and methods

### Study site and sample collection

Sediment samples were collected from water depths of 5437 to 10,954 m in the Mariana Trench (11° 20′ N, 142° 11.5′ E) on board of the 15th expedition of the Discovery-One research vessel (TS 15) in November 2019. A box corer was used to collect four sediment samples at depths of 5437, 6477, 7332 and 10,954 m in the Mariana Trench (Table 1). To prevent contamination, the sealed box corer was used during the sampling process. Sterilized shovels were used to scrape off the surface sediments (ca. 5 cm) of the box cores. All the parts of the samples that touched the sampler were discarded. The center part of the sediment core was subsampled and stored in sterile plastic bags immediately at 4 °C for subsequent experiments. In the laboratory, the sediment samples were scraped with a sterilized spoon and placed in a sterile centrifuge tube for subsequent fungal isolation experiments.

**Table 1:** Information for sediment samples used in this study.

Sample	Location	Water depth (m)	Sediment depth (cm)*
A	11.327°N, 142.188°E	10,954	0–18
B	10.761°N, 142.274°E	5437	0–10
C	10.761°N, 142.274°E	5437	10–20
D	10.942°N, 141.768°E	7332	0–10
E	10.942°N, 141.768°E	7332	60–70
F	10.813°N, 141.180°E	6477	0–10
G	10.813°N, 141.180°E	6477	30–40

The samples are named A–G.

*Approximate depth, as these samples were from subsampling of box cores.

### Fungal isolation and identification

The method of isolating and culturing fungi from deep-sea sediments was the same as the method of isolating bacteria^16^. An aliquot of sediment from the central part of the subsection was removed with a sterile spatula and placed in sterile vials for isolation^16^. Unless otherwise specified, Potato Dextrose Agar (PDA, 20% peeled and sliced potato, 1.0% glucose, and 1.5–2% agar, with deep-sea in-situ seawater, natural pH) was the routine media for the fungi. About 5 g of the sediment samples were placed in a 50 ml sterile centrifuge tube. 45 ml of deep-sea in-situ seawater which has been autoclaved were added into the tube, and mixed well. The sample was evenly spread on a PDA plate (100 ng/ml gentamicin or 100 ng/ml ampicillin), and then cultured at 28 ℃ for 7 days. The hyphaend-purification skill was used to isolate and purify the fungi. The individual strains were cultured on PDA medium at 28 ˚C. Hyphal growth and pigment production were observed and photographed every day. The Nikon DS-Ri2 was used to check hyphal growth and spore production. The species identification of fungi was carried out by combining morphological characteristics and internal transcription interval (ITS) sequences analysis.

### DNA extraction, PCR amplification, and clone library construction

The fungi were cultured in potato dextrose broth (PDB, 20% peeled and sliced potato and 1.0% glucose, with deep-sea in-situ seawater, natural pH) for 7–14 days for DNA isolation. Fungal genomic DNA was extracted from all targeted fungal strains using the TIANcombi DNA Lyse & Det PCR Kit (TIANGEN BIOTECH (BEIJING) CO., LTD). A partial region of 18S rDNA (nearly full length ITS sequences) was amplified by polymerase chain reaction with the primers ITS1 (5′-TCCGTAGGTGAACCTGCGG-3′) and ITS4 (5′- TCCTCCGCTTATTGATATGC-3′)^23^. Polymerase chain reaction mixture (20 μl) was composed of 10 μl 2 × Det PCR MasterMix, with 0.5 μl forward primer and 0.5 μl reverse primer, DNA template, and dd H_2_O. The PCR amplification process was as follows: starting with 3 min of denaturation at 95 °C, followed by 35 cycles of PCR (30 s at 95 °C, 30 s at 55 °C, and then 1 min at 72 °C), prolonged at 72 °C for 5 min. The amplified DNA sequences were analyzed by GENEWIZ for ITS sequencing.

### Phylogenetic analyses

The sequencing results were blasted in the NCBI database to determine the taxonomy of the isolates. All of the vector sequences from the sequenced fungal clones were analyzed using the rRNA Database Project CHECK CHI MERA program to detect and eliminate the potential chimeric sequences^24^. Pairwise alignment of the sequences was conducted using Clustal W in the MEGA7 software. Conserved motifs were identified, and the sequences were trimmed manually^25^. More than 98% of similar species were constructed into evolutionary trees to explore the species of the obtained strains.

### High-pressure cultivation and morphological observation

To observe if the fungal spores still have germination activity after the treatment of preset pressure, high-pressure tolerant assay was performed. The above-isolated strains were inoculated in the PDB at 28 °C for 10 days. Then fungal cultures were filtered with 8-layer sterile gauze to separate the hyphae and spores. The numbers of spores inoculated for each sample were shown in Table 2. A syringe was used to pick up 1 ml of spore filtrate of each strain as the targeted samples. The prepared samples were incubated for one week at a preset pressure, 0.1, 20, or 40 MPa. Once finished, 100 μl culture medium was re-inoculated on PDA medium supplemented with 100 μg/ml ampicillin (AMP) and then cultured at 28 ℃ under atmospheric pressure. Hyphal growth and colony morphology of the fungi were observed and recorded every day. This procedure was repeated for all the sediment samples.

**Table 2:** The initial inoculation amounts of each strain for high-pressure cultivation.

Sample	CIEL 1	CIEL 2	CIEL 3	CIEL 4	CIEL 5	CIEL 6	CIEL 7	CIEL 8
/ml	15	4.5 × 10^5^	6.5 × 10^5^	1.0 × 10^5^	70	20	1.0 × 10^5^	2.5 × 10^5^

For microscopy observation, 20 μl of culture was dropped in the center of the glass slide, mixed with 20 μl of Calcium Fluorescent white stain (Sigma-Aldrich) in the dark for 1 min. The microscopic morphology of the hyphae and spores of individual strains were observed under a fluorescence microscope (Nikon DS-Ri2) with a magnification of × 100 to × 400 times of ultraviolet light.

### Secondary metabolites

To determine the secondary metabolites produced by hadal fungi under different pressures, the fermentation and extraction of the fungi were performed. The fungal inoculation process, growth, culture conditions, and extraction of secondary metabolites were performed using the method as described in a previous study with minor modifications^26^. The 8 individual fungi were cultured under the three different pressures for one week. Then the same amounts of spores of each targeted strain were inoculated into 200 ml PDB medium, shaken at 180 rpm, 28 °C, for 14 days. The mycelium and liquid were separated by vacuum filtration with 8 layers of sterilized gauze, then the two parts were extracted twice with an equal volume of ethyl acetate overnight^27^. The organic fractions were collected and combined. The solvent was then removed by vacuum rotary evaporator at 45 °C. The obtained crude extract was dissolved in 1 ml methanol for subsequent analysis.

### UPLC-MS analysis

LC-Mass spectrometric analyses were performed using a Vanquish UPLC high-resolution mass spectrometer (Thermo Fisher) equipped with an electrospray ionization (ESI) source operating in the positive ion and negative mode. A Waters ACQUITY UPLC BEH C18 column (1.7 μm × 2.1 mm × 100 mm), at a flow rate of 0.4 ml/min, was used for preparative HPLC collection. The column temperature was held at 60 °C, and the injection temperature was held at 10 °C. The culture extracts were dissolved in 1 ml solvent (methanol: acetonitrile, 2:1, v/v) using a sonication bath for 5 min. Samples were centrifuged for 15 min and 400 μl of the supernatants were used for UPLC-MS analysis.

### Antimicrobial activity assay

The indicator bacteria used in the bacteriostatic circle experiment were provided by Shanghai Rainbowfish Company, including *Staphylococcus aureus* ATCC25923, *Enterococcus faecalis* FA2-2, *Escherichia coli* MG1655, *Chromobacterium violaceum* ATCC12472 CV026, *Candida Albicans desertification, mycobacterium smegmatis, Pseudomonas aeruginosa* PA01, *Pseudomonas aeruginosa* 484, *Pseudomonas aeruginosa* 554, *Pseudomonas aeruginosa* C218.

Antibacterial activity of the fungal extracts was performed using the standard disc diffusion method. The indicator bacteria culture solution (OD of approximately 0.5) was evenly spread on LB (Luria–Bertani, 5 g yeast extract, 10 g tryptone, 10 g sodium chloride, adjust the pH to 7.0), at an inoculum of 50 µl. The sterilized round filter paper sheets were attached to the LB. Then 6 μl of the crude extracted metabolites was dropped on the round filter paper sheet. Antibacterial assay was evaluated by measuring the diameters of inhibition zones, after incubated at 37 °C for 16 h. Empty solvent (ethyl acetate) was taken as a control for antibacterial activities. The areas of inhibition zones were measured using Image J, and repeated three times.

## Results

### Phylogeny of environmental fungal ITS-rDNA sequences

A total of 42 individual fungal strains were isolated from the Mariana Trench sediments in this study. High-pressure incubation and germination recovery of cultivable fungi were performed as described above. Through pressure screening, 8 sporulating filamentous fungi were selected as the targeted fungi for further analysis.

**Figure 1:**
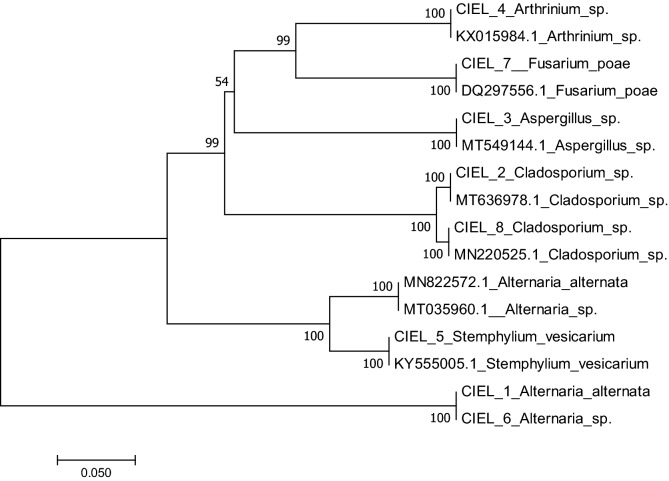
Phylogenetic tree of ITS sequences (ITS1/ITS4 primers) of the eight fungi isolated from deep-sea sediments in the Mariana Trench.

The phylogenetic tree was constructed by the MEGA7 software based on the ITS sequence (ITS1/ITS4 primer set) (Fig. 1). These 8 individual fungi belong to 6 different species, S*temphylium* sp., *Cladosporium* sp., *Arthrinium* sp., *Fusarium* sp., *Alternaria* sp., and *Aspergillus* sp. Although the blast sequences were 100% similar to those of known fungal strains in the database, only two of the 8 individual fungal were determined at species level while the other 6 were identified only at genus level. Moreover, the sequences of some individual fungi showed 100% similarity to two or more species in the database alignments. Therefore, identification was also carried out by the typical morphological taxonomy, combined with molecular identification. Based on these characterizations, the 8 fungi were named as *Alternaria alternata* CIEL 1*, Cladosporium* sp. CIEL 2*, Aspergillus* sp. CIEL 3*, Arthrinium* sp. CIEL 4*, Stemphylium vesicarium* CIEL 5, *Alternaria* sp. CIEL 6, *Fusarium poae* CIEL 7*,* and *Cladosporium* sp. CIEL 8 (Table 3). These results suggested the presence of cultivable fungal populations in the Mariana Trench sediments.

**Table 3:** Species information for hadal-derived fungi isolated and cultured in this experiment.

No	Name	GenBank accession no	Fungal genera or species	Sample (sediments)
CIEL 1	*Alternaria alternata* CIEL 1	MN822572.1	*Alternaria alternata*	B
CIEL 2	*Cladosporium* sp. CIEL 2	MT636978.1	*Cladosporium* sp.	D
CIEL 3	*Aspergillus* sp. CIEL 3	MT549144.1	*Aspergillus* sp.	C
CIEL 4	*Arthrinium* sp. CIEL 4	KX015984.1	*Arthrinium* sp.	F
CIEL 5	*Stemphylium vesicarium* CIEL 5	KY555005.1	*Stemphylium vesicarium*	D
CIEL 6	*Alternaria* sp. CIEL 6	MT035960.1	*Alternaria* sp.	G
CIEL 7	*Fusarium poae* CIEL 7	DQ297556.1	*Fusarium poae*	C
CIEL 8	*Cladosporium* sp. CIEL 8	MN220525.1	*Cladosporium* sp.	C

### Fungal characterization and identification

**Figure 2:**
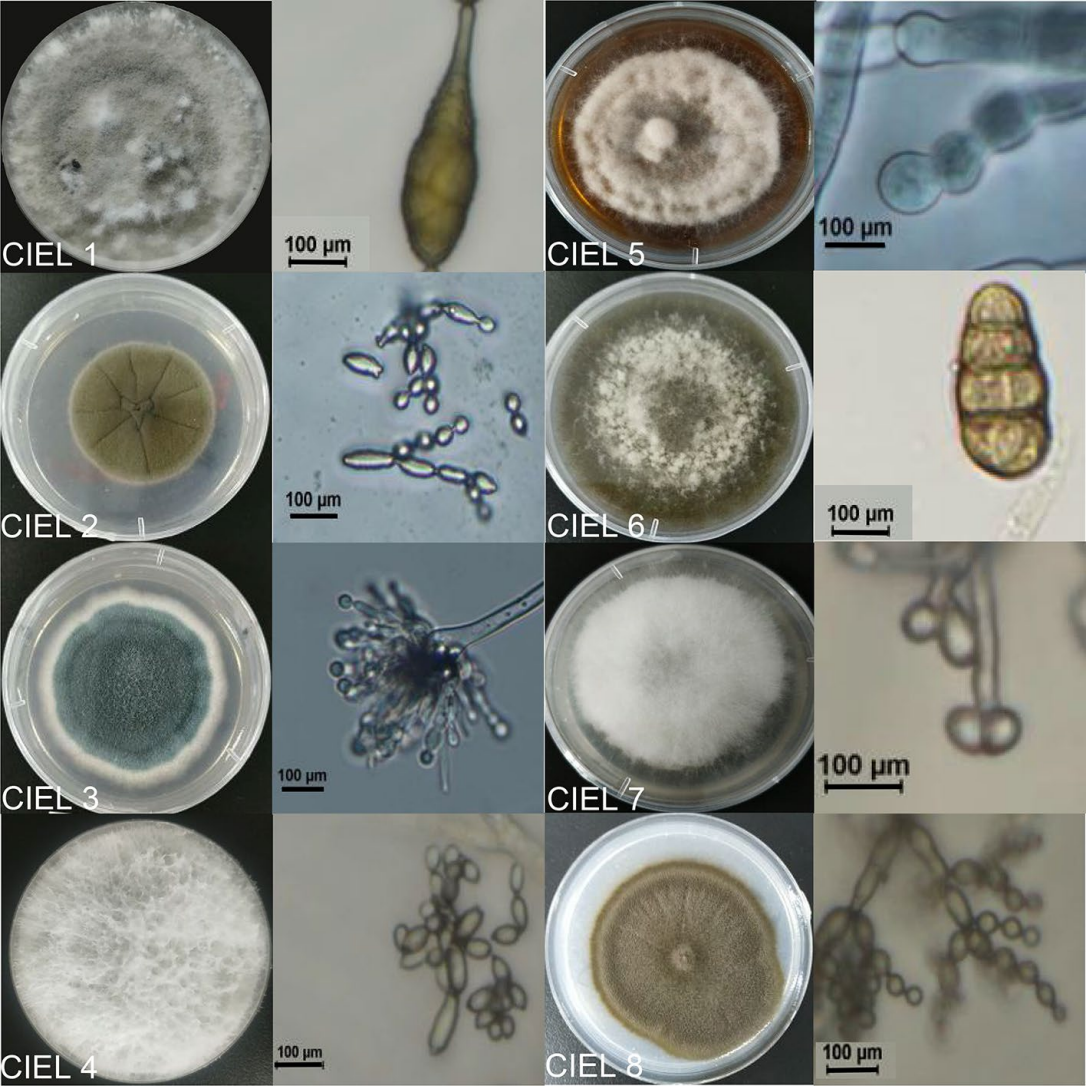
The typical spore patterns of 8 strains were obtained in the Mariana Trench sediment under atmospheric pressure. Macroscopic images showed the morphology of colony. Microscopic images showed the morphology of typical spores, taken under × 40 microscopes. The scale bar was 100 μm.

To characterize the isolated strains, morphological identifications of the 8 fungal colonies were performed, including microscopy observations. All the hyphae were permeable to the Calcofluor white (CFW) stain. *A. alternata* CIEL 1 was a filamentous fungus that produced a gray-white thick colony and finally turned dark brown to black. Under the microscope, the dark-brown spores appeared larger with fine longer septa. After 14 days on PDA at 28 ℃, the colony reached 6 cm diameter. Another species, *Alternaria* sp. CIEL 6, showed the typical characteristics of *Alternaria* sp., with distinctive septate and club-shaped spores. After 14 days on PDA at 28 ℃, the colony reached 6-cm diameter, and created the dark green pigment. The basal hyphae were pigmented under the microscopic view (Figs. 2, S1).

*Cladosporium* sp. CIEL 2 and *Cladosporium* sp. CIEL 8 belong to the same species. The two species developed brown colonies with smooth edges and wrinkled surface. Under the microscope, both species produced dark-pigmented spores and branched hyphae. The fascicled spores were ovoid, round, or irregularly shape. *Cladosporium* sp. CIEL 2 colony diameter reached 3–4 cm after 7 days on PDA at 28 ℃. But the diameter of *Cladosporium* sp. CIEL 8 colony reached 4–5 cm after 7 days on the same medium and temperature (Figs. 2, S1).

*Aspergillus* sp. CIEL 3 belongs to a common species widely distributed in different environments. It produced distinctive uniseriate and columnar conical heads with the phialides, under the microscope. After 7 days on PDA at 28 °C, the colony reached 4–5 cm diameter. The color of the colony was blue. The hyphae and scattered spherical conidiophores were colorless (Figs. 2, S1).

*F. poae* CIEL 7 was also isolated from sediment C, which belongs to a large genus of filamentous fungi. Here, after 7 days on PDA at 28 °C, the colony reached 4–5 cm diameter. It developed oval or spindle-shaped spores and colorless hyphae, extending in the branched mycelium. Conidiophores were short and loosely branched (Figs. 2, S1).

*Arthrinium* sp. CIEL4 grew fast and developed into a white, floccose, spreading colony, brown to black spore clusters with time. After 7 days on PDA at 28 ℃, the colony reached 6 cm in diameter. It produced small lemon- or olive-shaped spores with characteristic features, under the microscope (Figs. 2, S1).

*S. vesicarium* CIEL 5 matched 100% similarity to *S. vesicarium.* After 7 Days on PDA at 28 ℃, the colony reached 4–5 cm in diameter, and then there was no significant change in the colony diameter. *S. vesicarium* CIEL 5 developed a white colony and turned brown to tan in the center, secreting orange pigment into the medium. Spores were dark in color, few branching. Conidia were porous, monoculture at the distended end of the pedicel or spores were produced continuously at the tip (Figs. 2, S1).

### Hydrostatic pressure impacts the germination and vegetative growth of hadal sediment fungi

**Figure 3:**
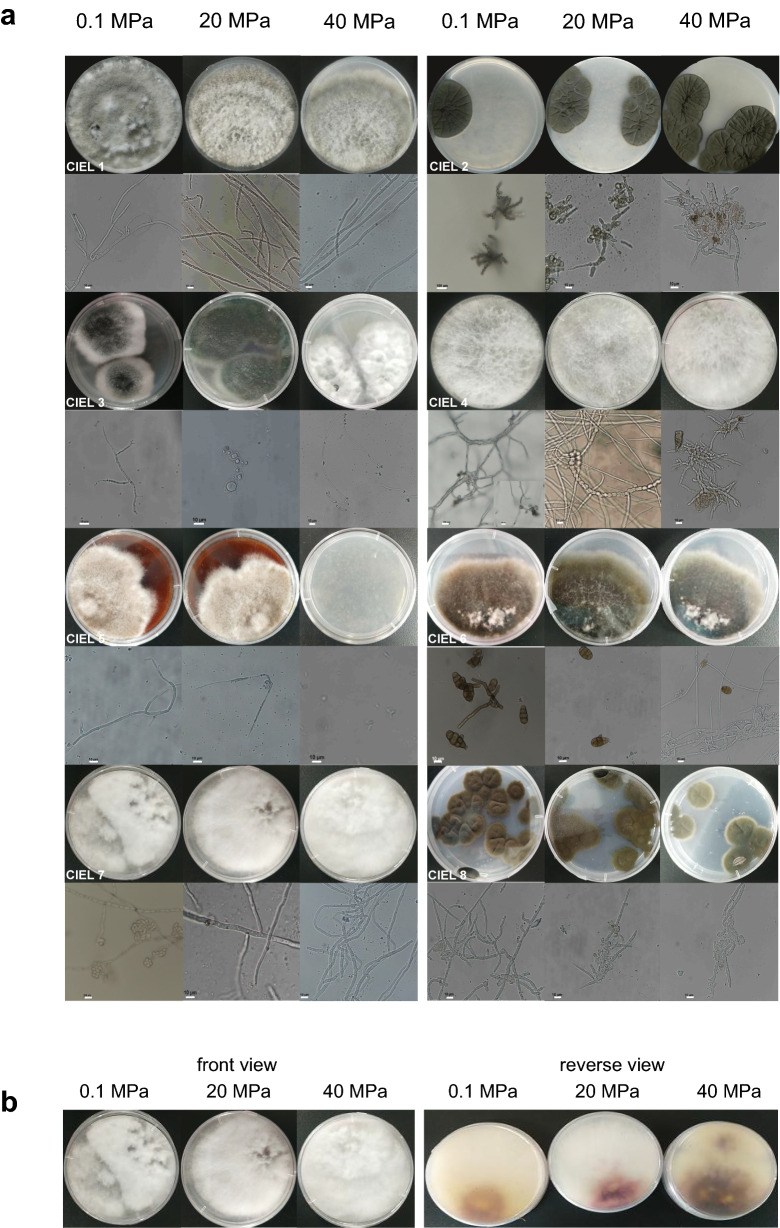
Phenotypes of hadal-derived fungi isolated and cultured in this experiment under the different preset pressures. (**a**) Colony, mycelium and spore morphology of eight fungi were obtained, respectively, under 0.1, 20 and 40 MPa. (CIEL 1) *Alternaria alternata* CIEL1, (CIEL 2) *Cladosporium* sp. CIEL 2, (CIEL 3) *Aspergillus* sp. CIEL 3, (CIEL 4) *Arthrinium* sp. CIEL 4, (CIEL 5) *Stemphylium vesicarium* CIEL 5, (CIEL 6) *Alternaria* sp. CIEL 6, (CIEL 7) *Fusarium. poae* CIEL 7, (CIEL 8) *Cladosporium* sp. CIEL 8. (**b**) Growth state of *Fusarium poae* CIEL 7 under 0.1, 20 and 40 MPa. There was no significant difference in the front side of the colonies, but the back side of the colonies showed different colors.

Among the 8 isolated hadal fungi, all except *S. vesicarium* CIEL 5 possessed the ability of germination after being incubated under the pressure of 40 MPa. The percentage of spores germination under different pressures was 20–50%. CIEL 5 did not germinate after 7 days’ high-pressure incubation and no mycelium was developed after another 7 days’ atmospheric-pressure cultivation (Fig. 3). The spore phenotypes of 8 hadal-sediment-derived fungi, cultured under different hydrostatic pressures were observed in this study. There was no obvious change of spores structure before and after HPP treatment (Fig. S2). Those results suggested that high-pressure impacts germination of these fungal species (CIEL 5), but no obvious effect on the phenotype of fungal spores.

**Figure 4:**
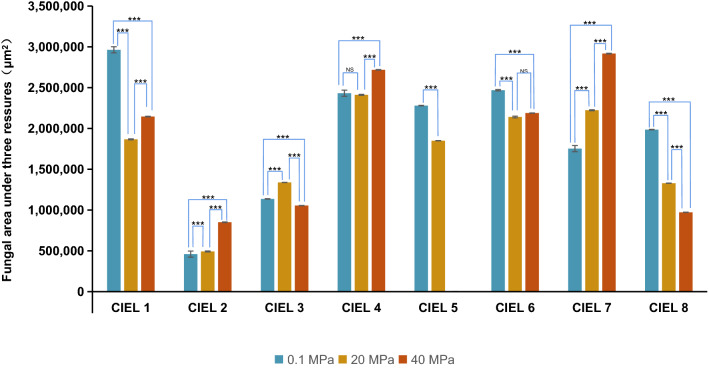
The growth rate of hadal-derived fungi isolated and cultured in this experiment under the different preset pressures. Under the same cultural conditions, the area of the strain changed regularly with the change of pressure. The unit of the area was μm^2^. T-test was used for pairwise comparisons. (NS *p* > 0.05, ***p < 0.001).

Next, we performed phenotype characterization and measured colony areas of the fungal strains cultured under different pressures. Among the eight individual fungi, some of the fungal colonies developed significantly larger areas on the plates under atmospheric pressure than under high pressures, such as CIEL 1, CIEL 5, CIEL 6, and CIEL 8, while other fungi grew better under high pressure, such as CIEL 2, CIEL 3, CIEL 4, and CIEL 7 (Fig. 4). Coloration of these fungi was also influenced by hydrostatic pressure. The fungal colonies of CIEL 3 and CIEL 7 developed distinct phenotypes under different pressures. Colonies of CIEL 3 were dark-green under atmospheric pressure, and green under 20 MPa. The pigment produced by CIEL 7 was yellowish under normal pressure, purple-red under 20 MPa, and blackish under 40 MPa (Fig. 3). Based on these results, we conclude that high hydrostatic pressure influences germination, vegetative growth, and pigmentation of the Mariana Trench sediment- derived fungi.

### High hydrostatic pressure influences the production of secondary metabolites

**Figure 5:**
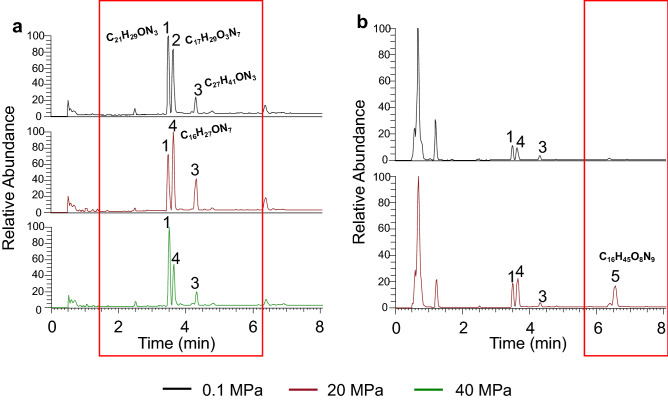
TIC diagrams of metabolites of hadal-derived fungi isolated and cultured under three preset hydrostatic pressures. (**a**) TIC peak of *Cladosporium sp.* CIEL 2 at different hydrostatic pressures. In addition to the change of peak intensity, 4 but not 2 appeared in the metabolites at 20 MPa and 40 MPa. (**b**) TIC peak of *Stemphylium vesicarium* CIEL 5 at different hydrostatic pressures. A new peak 5 appears at 20 MPa. Each analysis shows the extracted ion track of the corresponding compounds recorded by mass spectrometry. The relative abundance was set at 100.

In this study, the secondary metabolites of the 8 individual fungi were extracted and characterized. To better understand the impact of hydrostatic pressure on the production of secondary metabolites, the UPLC-MS experiment was performed. In the comparison of metabolites from the same strain under different hydrostatic pressures, different mass peaks in the total ion chromatogram (TIC) were observed (Fig. 5). For CIEL 2, the amounts of compound 1, 2/4, 3 with retention times (RT) at 3.49, 3.65, 4.32 min, were changed respectively because of the treatment pressure. Through blasting in the NIST chemical database, the compounds were identified as C_21_H_29_ON_3_, C_17_H_29_O_3_N_7_/ C_16_H_27_ON_7_, and C_27_H_41_ON_3_, respectively. In addition, as shown in Fig. S3, compounds 2 and 4 had the same retention time, but not one compound. The new peak 5 (RT at 6.6 min) was observed in the TIC of CIEL 5 after high pressure treatment at 20 MPa. The putative compound was identified as C_16_H_45_O_8_N_9_. Further characterization is needed to determine the exact chemical structure of these compounds. Our results indicated that fungal growth pressure probably affected expression of genes associated with biosynthesis of these compounds. Further, the production of secondary metabolites changed with fungal growth pressure. This hypothesis was further tested by antimicrobial assay.

### The antibacterial bioactivity of secondary metabolites and the influences of hydrostatic pressure

**Figure 6:**
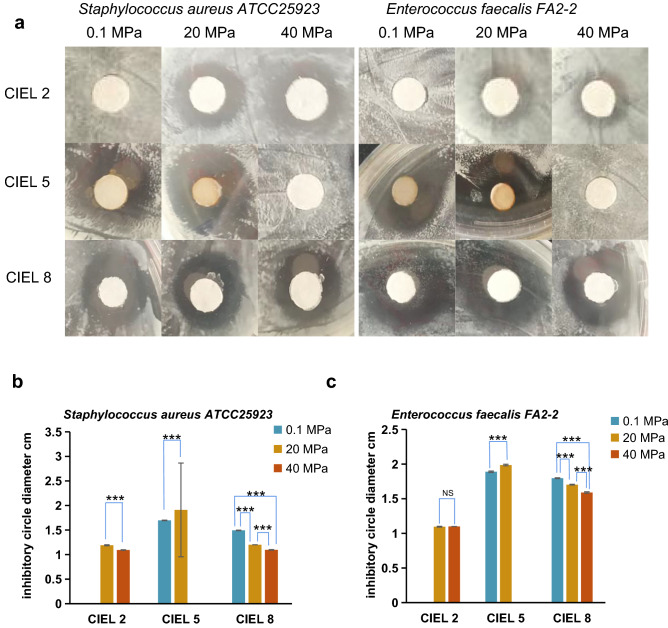
Antimicrobial activity of metabolites of hadal-derived fungi isolated and cultured under three preset hydrostatic pressures. Among the eight strains of fungi isolated in this experiment, Antimicrobial activity of metabolites produced by *Cladosporium* sp. CIEL 2, *Stemphylium vesicarium* CIEL 5 and *Cladosporium* sp*.* CIEL 8 against *Staphylococcus aureus* ATCC25923 and *Enterococcus faecalis* FA2-2 under 0.1, 20 and 40 MPa. (**a**) The inhibitory zone of metabolites produced by fungi under different pressures. (**b**) Antimicrobial zone diameter of the metabolites against *S. aur*eus ATCC25923. The diameter unit was cm. T-test was used for pairwise comparisons. The activity of the *Stemphylium vesicarium* CIEL 5 metabolic products increases with the increase of pressure. However, the activity of the *Cladosporium* sp. CIEL 2 and *Cladosporium* sp*.* CIEL 8 metabolic products decreases with the increase of pressure. (***p < 0.001). (**c**) Antimicrobial zone diameter of the metabolites against *E. faecalis* FA2-2. The diameter unit was cm. T-test was used for pairwise comparisons. *Cladosporium* sp. CIEL 2 metabolic products are inactive, the activity of the *Stemphylium vesicarium* CIEL 5 metabolic products increases with the increase of pressure, and the activity of the *Cladosporium* sp*.* CIEL 8 metabolic products decreases with the increase of pressure (NS *p* > 0.05, ***p < 0.001).

To determine the bioactivity of these natural products, antimicrobial assay was performed against 10 pathogenic bacteria via the Kirby-Bauer test. Among the 8 fungal strains, *Cladosporium* sp. CIEL 2, *S. vesicarium* CIEL 5, and *Cladosporium* sp. CIEL 8 displayed antibacterial activity against *S. aureus* ATCC25923 and *E. faecalis* FA2-2*.* The diameters of the inhibition zones were measured as shown in Fig. 6. First, the extract of *Cladosporium* sp. CIEL 2 cultured under atmospheric pressure showed no antibacterial activity. However, the extracts from the same strain (CIEL 2) incubated under 20 MPa and 40 MPa exhibited remarkably high inhibition effects on *S. aureus* ATCC25923 and *E. faecalis* FA2-2. Second, *S. vesicarium* CIEL 5, cultured under 0.1 MPa and 20 MPa, produced brownish pigments in the crude extract, and lost the ability of germination at 40 MPa. Compared with other fungal metabolites, the extracts of CIEL 5 cultured under 0.1 MPa and 20 MPa had the most significant antibacterial effects on *S. aureus* ATCC25923 and *E. faecalis* FA2-2. Third, secondary metabolites produced by *Cladosporium* sp. CIEL 8 under the three pressures all showed inhibition activities against both *S. aureus* ATCC25923 and *E. faecalis* FA2-2. The size of the inhibition zones appeared smaller gradually as growth pressure increased (Fig. 6). Our results showed the vast potential of the hadal fungi to be exploited as the producer of bioactive compounds. Furthermore, it was assumed that the genes associated with biosynthesis of secondary metabolites could be activated by the high-pressure treatment, implying the HHP influenced the production of natural products and life process of the Mariana Trench-derived fungi.

## Discussion

In our report, six species of *Alternaria* sp., *Cladosporium* sp., *Aspergillus* sp., *Arthrinium* sp., *Stemphylium* sp., and *Fusarium* sp. isolated from the Mariana Trench sediments are reported for the first time. Prior to this report, sediment fungal communities were mostly estimated by metagenomic analysis (culture-independent method), showing the distinct diversity of microorganisms and genetic potential^17,28,29^. Here, our report of isolation of over 40 individual fungal strains will facilitate the development of new biological sources of natural products. Furthermore, the Mariana Trench sediment-derived fungi offer excellent model organisms to study the physiology of HHP-adapted fungi and understand their ecological roles in such extreme environments.

Using classical ITS and morphology-based identification, most of the isolated fungal species were similar to terrestrial fungal species and those derived from the shallow sea. Furthermore, most of the fungi isolated in our study exhibited phylogenetic similarity to the pathogen fungi in the environment. For example, *S. vesicarium* has wide host range as a plant pathogen, including asparagus, garlic, and pear. Many species of *Cladosporium* are commonly found on living and dead plant material. Some of them are plant pathogens, others can parasitize other fungi. *Arthrinium phyllostachium* sp. was isolated from the decaying culms of *Phyllostachys heteroclada*^30^. *Fusarium poae* can cause brown necrosis of petals and, sometimes, the death of opening buds. *Alternaria alternata* is ubiquitous worldwide and can cause black spots in many fruits and vegetables around the world. *Aspergillus sydowii* is known to cause infection/death in the coral community, also a causative agent for aspergillosis, onychomycosis, and keratomycosis. From these results, we posit that the hadal sediment fungi were probably derived from spores or appressorium of terrestrial fungal strains, which were transported with their host (plants), in the prior time. Then the fungal species from the terrestrial environment have gradually adapted to the extreme trench environment. Further work is needed to verify the origin of the hadal fungi.

Although the adaptation mechanism of piezophilic bacteria in deep-sea environments was well recognized in many reports, the investigations on the adaption of sediment fungi to high hydrostatic pressure of hadal trenches have received very little attention. In our study, most of the isolated fungi germinated and grew normally after the incubation under elevated hydrostatic pressure for 7 days. Only *S. vesicarium* CIEL 5 lost the ability of germination after the treatment of high hydrostatic pressure (40 MPa). Damare and Raghukumar^18^ showed the abnormal phenotype and different biomass of deep-sea fungi under high pressure (30 MPa), compared with that at atmospheric pressure. They reported all deep-sea fungi grow better at 20 MPa and 5 °C than that cultured at atmospheric pressure in their study^18^. In this study, the Mariana Trench sediment fungi showed different growth rates at 0.1, 20, and 40 MPa. Some cultures showed better growth at higher pressure while others preferred atmospheric pressure for growth. We also showed different phenotypes and coloration under different pressures. It seems that the fungi evolved into distinct phenotypes, in order to adapt to the extreme environment. The fungal vegetative growth was affected by high hydrostatic pressure, demonstrating that the extreme environmental condition shapes the development of hadal fungi.

Many bioactive natural compounds from deep-sea fungi have been reported. The extreme and distinct growing environments breed the particular secondary metabolisms, biosynthesizing novel natural products^19,31,32^. The deepest sediment harbors the most interesting natural compounds. But most researchers analyze the natural products of deep-sea fungi under laboratory conditions, losing the distinctiveness. Here we reported that the productions of secondary metabolites of the fungi were influenced by the pressure condition. Half of the 8 hadal sediment-derived fungi produced antibacterial compounds. The amounts of the bioactive compounds were influenced by growth pressures, indicating that growth pressure plays a role in biosynthesis of secondary metabolites. The distinct pigment production of cultures under different pressures also corroborated the adaptive hypothesis. To our knowledge, the treasure trove of hadal fungi is almost unknown, not to mention the secondary metabolites produced in situ high-pressure cultivation. Only a few studies have reported the natural products of hadal fungi in the lab condition (at atmospheric pressure), lacking in the investigation of ecological adaptation and production of novel secondary metabolites^12^. Our results contribute to paving the way for more exploration and in-depth investigation of specific secondary metabolism in the extreme environment and the relationship between the production of natural products and the environment.

In conclusion, there are few studies on the adaptation mechanisms of deep-sea fungi, especially those living in hadal trenches, and the cognitive exploration of their life processes and genetic resources are extremely limited^33,34^. Therefore, our study of the fungi derived from the Mariana Trench sediments not only contributes to the exploitation of unknown microbial resources in the deep sea, but also has important theoretical significance for the exploration of deep-sea life processes and evolutionary mechanisms, providing a basis for human exploitation and utilization of the biological resources in the Mariana Trench.

## Supplementary Information

Supplementary Figures.

## Data Availability

All data generated or analysed during this study are included in this published article (and its Supplementary Information files).The datasets supporting the conclusions of this article are available in the NCBI database, GenBank accession numbers list as follows: MZ156971 (CIEL-1), MZ156972 (CIEL-2), MZ156974 (CIEL-3), MZ156973 (CIEL-4), MZ156978 (CIEL-5), MZ156979 (CIEL-6) MZ156981 (CIEL-7), MZ156980 (CIEL-8).
