# Supplementary material for: High hydrostatic pressure shapes the development and production of secondary metabolites of Mariana Trench sediment fungi
Source: Sci Rep. 2021 Jun 1;11:11436. doi: 10.1038/s41598-021-90920-1 (PMC8169743; doi:10.1038/s41598-021-90920-1)
Supplement: Supplementary file 1 — Supplementary Figures. [file 41598_2021_90920_MOESM1_ESM.pdf]

High hydrostatic pressure shapes the development and production of secondary metabolites of Mariana Trench Sediment Fungi

Qingqing Peng, Yongqi Li, Ludan Deng, Jiasong Fang and Xi Yu*

running head: Hadal sediment fungi and bioactive secondary metabolites

Address:

Shanghai Engineering Research Center of Hadal Science and Technology,
College of Marine Sciences, Shanghai Ocean University, Shanghai, China

* corresponding author XI YU (Email: xyu@shou.edu.cn)

Key words: Piezotolerance, Mariana Trench, fungi, secondary metabolites

Figure S1

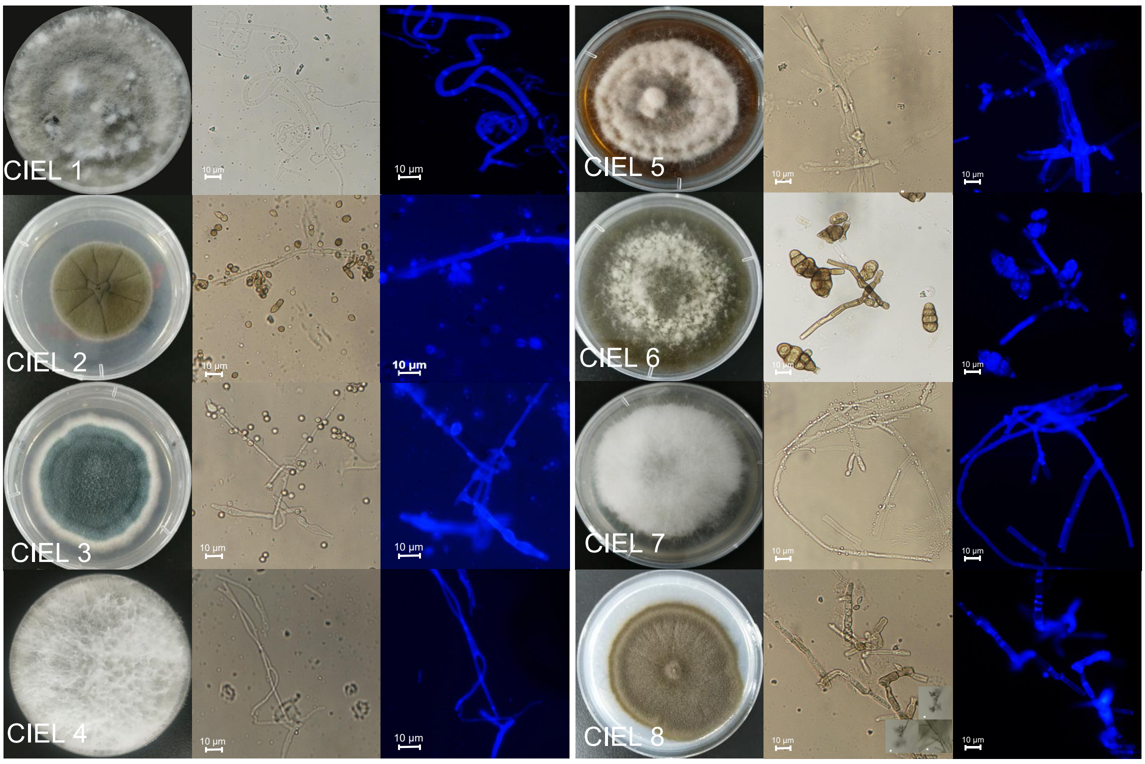

Supplementary Figure S1. The phenotypes of 8 fungal strains obtained in this experiment. Macroscopic and microscopic images of 8 fungi isolated from the Mariana Trench sediments under atmospheric pressure. Macroscopic images showed the phenotype of colony. Microscopic images showed the morphology of mycelium and spores, taken under × 40 microscopes / Fluorescent Microscope. The scale bar was 10 μm.

Figure S2

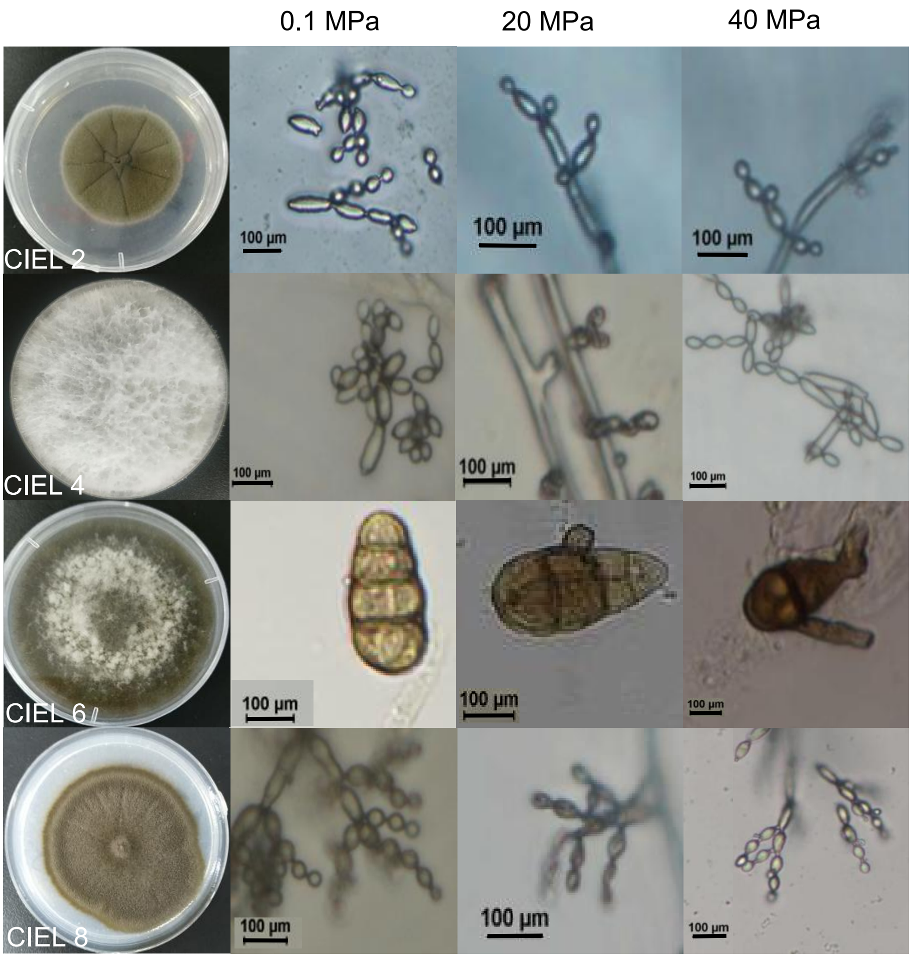

Supplementary Figure S2. The spores phenotypes of 8 hadal-sediment-derived fungi, cultured under different hydrostatic pressures, were observed in this study. There wes no obvious change of spores structure before and after HPP treatment. So the spores phenotypes of 4 strains were selected to show here as representative. Macroscopic images showed the phenotype of colony. Microscopic images showed the morphology of spores, under ×40 microscope. The scale bar was 100 μm. Hydrostatic pressure had no obvious effect on fungal spore phenotype.

Figure S3

a

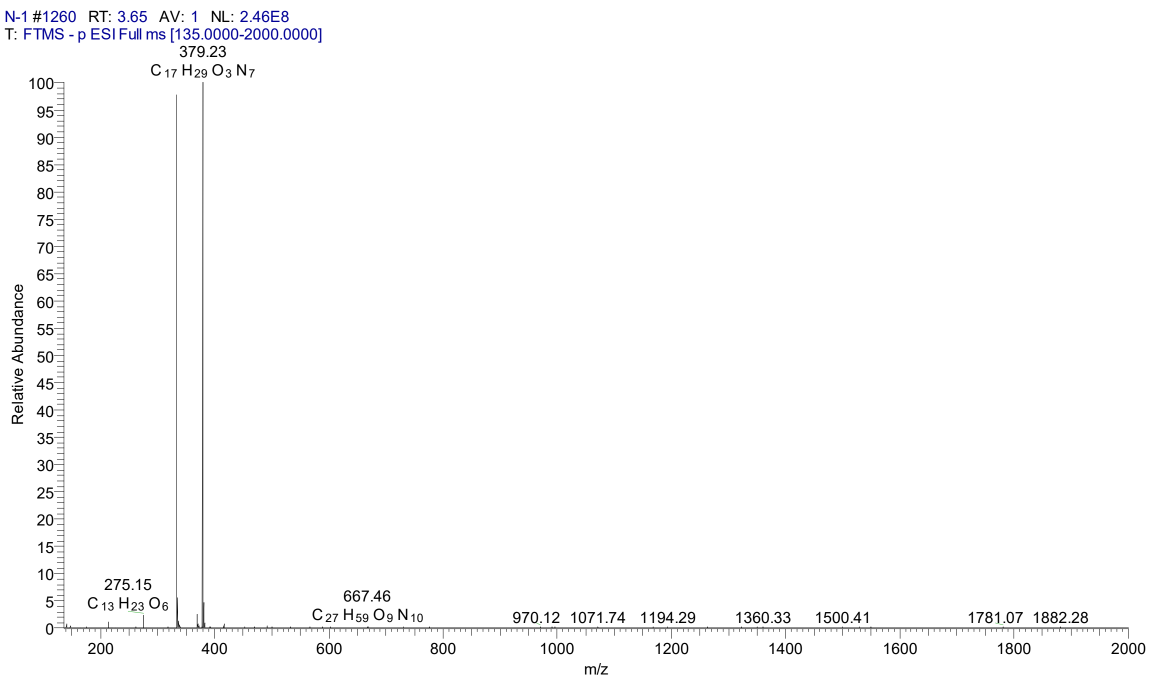

b

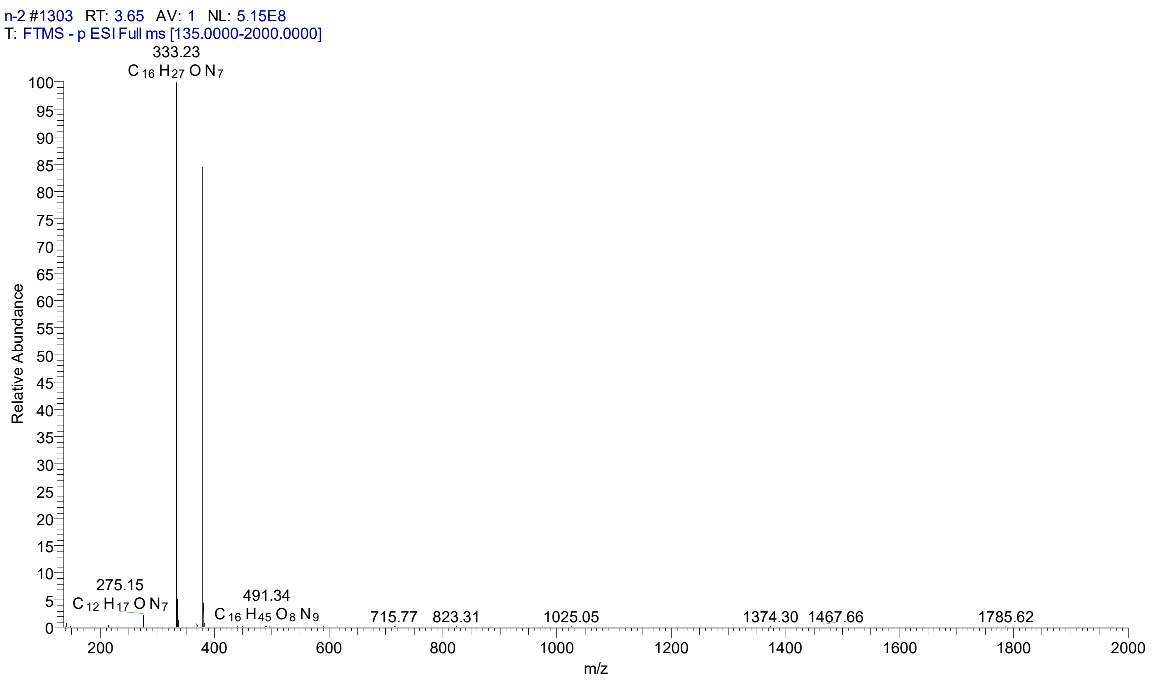

Supplementary Figure S3. The mass spectrum data of compounds 2 and 4 in Fig. 5(a). The figure showed that compounds 2 (a) and 4 (b), which had similar retention times, were not the same compound.
